# Association between Accelerometry and Incident Dementia in the National Healthy Aging Trends Study (NHATS)

**DOI:** 10.1002/alz70860_097723

**Published:** 2025-12-23

**Authors:** Matthew Ko, Shirley Huang, Karl Brown, Andrew Shutes‐David, Katherine Wilson, Edmund Seto, Debby W Tsuang

**Affiliations:** ^1^ University of St Andrews, St Andrews, Fife, United Kingdom; ^2^ University of Washington, Seattle, WA, USA; ^3^ Geriatric Research, Education, and Clinical Center, Seattle, WA, USA; ^4^ Mental Illness Research, Education, and Clinical Center, VA Puget Sound Health Care System, Seattle, WA, USA; ^5^ Geriatric Research, Education, and Clinical Center, VA Puget Sound Health Care System, Seattle, WA, USA; ^6^ VA Puget Sound, Seattle, WA, USA

## Abstract

**Background:**

Elderly individuals are vulnerable and at higher risks of developing dementia than younger adults. While there are many associated co‐morbidities that can exacerbate dementia status, little is known about how physical activity measured via accelerometry is associated with the development of dementia.

**Method:**

Wrist‐worn accelerometry collected on nationally representative sample of 562 individuals aged 65 years and older from the NHATS were used to assess the association between actigraphy measures and incident dementia, as determined by cognitive tests.

**Result:**

Physical activity measured by actigraphy was associated with lower risk of incident dementia (OR = 0.66, 95% CI [0.55 ‐ 0.79]), including in analyses adjusting for age, gender, and comorbidities (OR = 0.68, 95% CI [0.57 ‐ 0.82]). Receiver operating characteristic (ROC) curves indicate that accelerometry performs slightly better than self‐reported physical capacity as a measure of incident dementia risk.

**Conclusion:**

Wrist‐worn accelerometry is feasible, and physical activity is found to be significantly associated with lower risk dementia in the NHATS. These accelerometry findings add to the evidence that physical activity may reduce cognitive impairment and dementia risk.

